# Supplementary material for: Ising meson spectroscopy on a noisy digital quantum simulator
Source: Nat Commun. 2024 Jul 13;15:5901. doi: 10.1038/s41467-024-50206-2 (PMC11246482; doi:10.1038/s41467-024-50206-2)
Supplement: Supplementary file 1 — Supplementary Information [file 41467_2024_50206_MOESM1_ESM.pdf]

Supplementary Materials:
Ising Meson Spectroscopy on a Noisy Digital Quantum Simulator

Christopher Lamb,[1,*] Yicheng Tang,[1] Robert Davis,[1] and Ananda Roy[1,†]

[1] *Department of Physics and Astronomy, Rutgers University, Piscataway, NJ 08854-8019 USA*

The supplementary material is organized as follows. In Sec. I, details of the experimental implementation are provided together with additional data used in the main text of the manuscript. Sec. I C outlines the experiment protocol to execute the XYZ chain with transverse and longitudinal fields, followed by Sec. I D for the XY chain in the presence of a longitudinal field. Only noiseless Qiskit simulations and comparison with exact computations are provided for the XY model.

I. SUPPLEMENTARY INFORMATION: SUPPLEMENTARY NOTES

The quench protocol was performed using both pulse-scaled and non-pulse-scaled circuits.

A. Pulse Scaled Experiment

The experiment is set up by initializing the $|0,...,0\rangle$ ground state, and a unitary comprised of the trotterized time evolution of the Hamiltonian is applied for each Δt. After each Trotter step, the desired observable is measured. For more details, see the Methods section of the main text.

The magnetization was calculated by taking the quasi-probabilities and converting them to the binary probabilities, then using counts to get the magnetization for each of the measured qubits for each time step. This experiment was executed five times, each with 8,192 shots, and the results of the average magnetization is shown in Supplementary Figure 2. To find the meson rest energies, we took the absolute value squared of the Fourier transform of the average magnetization to find the local maxima shown in Supplementary Figure 3. The simulation where $g = 1.0$ was also executed with the 20-qubit loop, and we found the result consistent with the 12-qubit loop (see Supplementary Figure 4).

B. Non-Pulse Scaled Experiment

The experiment was initialized to $|\psi\rangle = |\rightarrow\rangle^{\otimes L}$ by applying a Hadamard gate to each qubit in the $|0\rangle^{\otimes L}$ ground state. The unitary applied for the trotterized time evolution was constructed the same way as the previous experiment

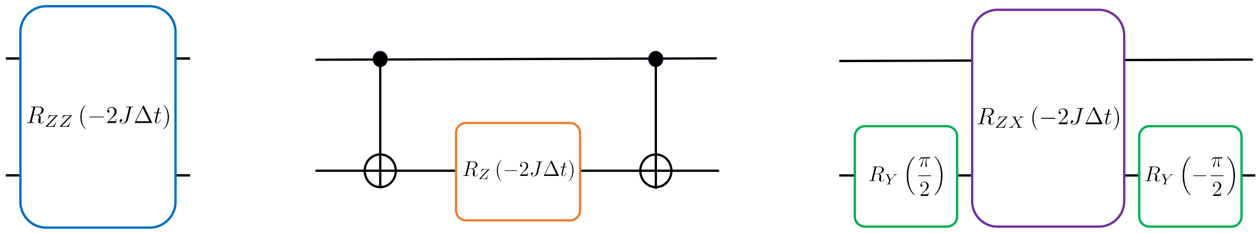

(a) R_{zz} gate (b) R_{zz} gate transpiled into basis gates (c) Pulse-efficient equivalent to R_{zz}

Supplementary Figure 1: a) Gate conversion breakdown of the R_{zz} gate. b) Transpilation of R_{zz} into the basis gates $CNOT$ and R_z. Each of the two $CNOT$ gates in the transpiled R_{zz} represents a $R_{zx}(\pi/2)$ rotation implemented by an echoed cross-resonance (CR) pulse. c) Convertion of the R_{zz} gates in our unitary to a R_{zx} gate with R_y rotations. This leads to the area of the CR pulse being scaled down. This gate change reduces the compounding errors of our unitary.

* cdl92@physics.rutgers.edu
† ananda.roy@physics.rutgers.edu

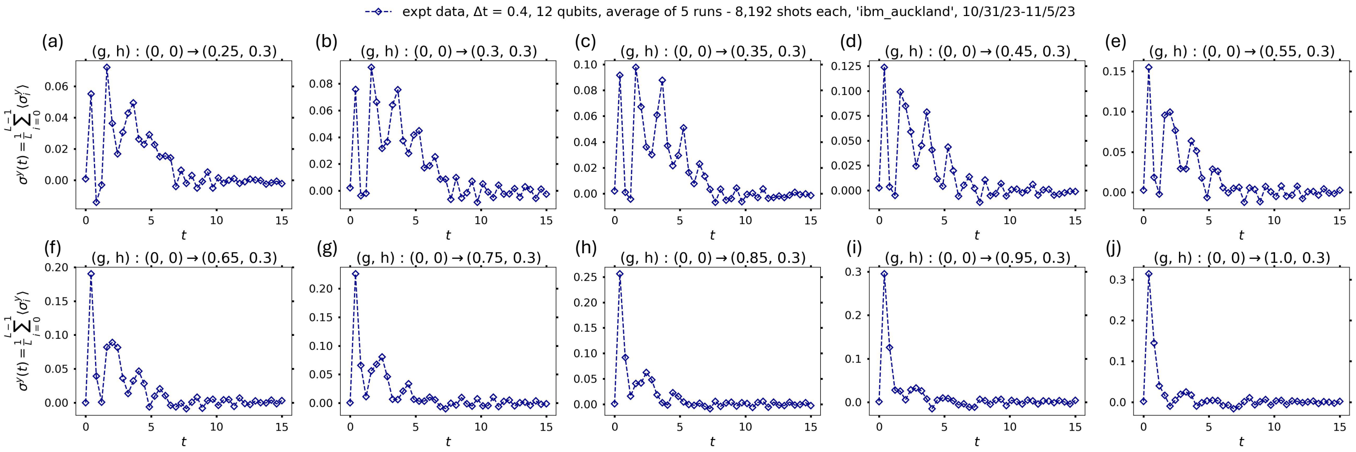

Supplementary Figure 2: Experimental results for the measurement of $\sigma^y(t)$ [panels a) - j)] for quenches from $g = h = 0$. The results are an average of 5 experimental runs, each with 8,192 shots. The Qiskit M3 error mitigation and dynamical decoupling schemes were used to mitigate the effects of noise present in the IBM Auckland simulator. These data are analyzed for Fig. 2 and 3 of the main text (see corresponding figures for further details). Dashed blue lines are meant to be a guide for the eye.

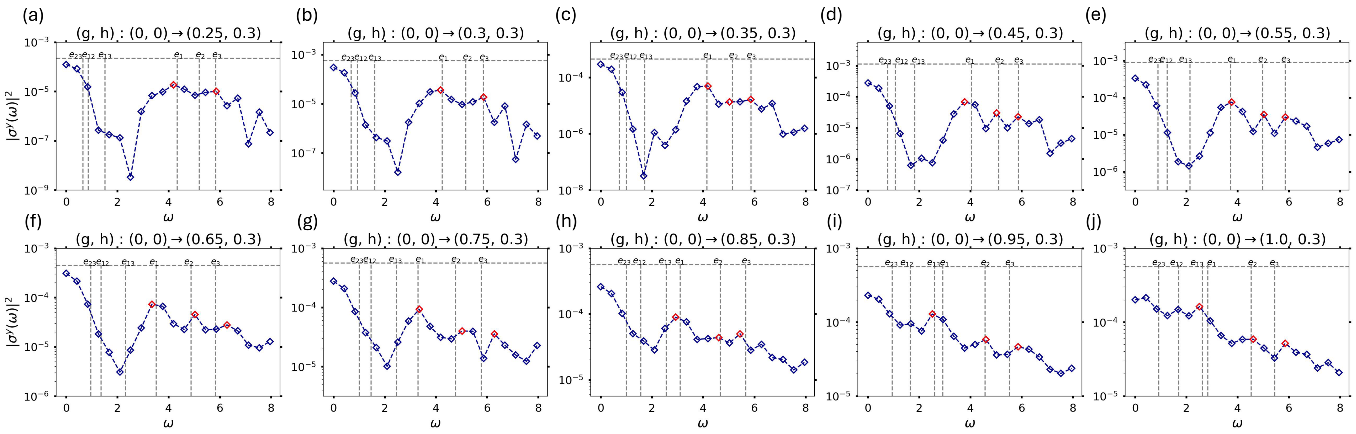

Supplementary Figure 3: Absolute value of the Fourier transforms of the data shown in Fig. 2 [panels a) - j)]. The different meson rest energies are obtained by locating the maxima in the corresponding data (red markers). By locating the local maxima, error bars were added to Figure 3 of the main text with the values of $2\pi/t$ where $t = 15$. Dashed blue lines are a guide for the eye.

for the Hamiltonian:

$$H = -\sum_{j=0}^{L-1} (\sigma_j^x \sigma_{j+1}^x + g\sigma_j^z + h\sigma_j^x) \tag{1}$$

The implementation of this experiment on `ibmq_mumbai` is similar to the non-pulse scaled experiment. The circuits were transpiled to map the qubits of the circuit to the qubit loops available on the connectivity graph. Circuits were executed using the qiskit runtime primitive, Sampler. Dynamical decoupling is added when the circuits are transpiled, and Matrix-free Measurement Mitigation (M3) is used on the resulting counts.

When the results are returned, the magnetization is calculated by taking quasi-probabilities and converting them to binary probabilities. With the binary probabilities, marginal counts are used to get the magnetization of each qubit. The results are shown in Supplementary Figure 5 for one run executed on `ibmq_mumbai` with 100,000 shots. To find the meson rest energies, we took the absolute value squared of the Fourier transform of the average magnetization to find the local maxima shown in Figure 6.

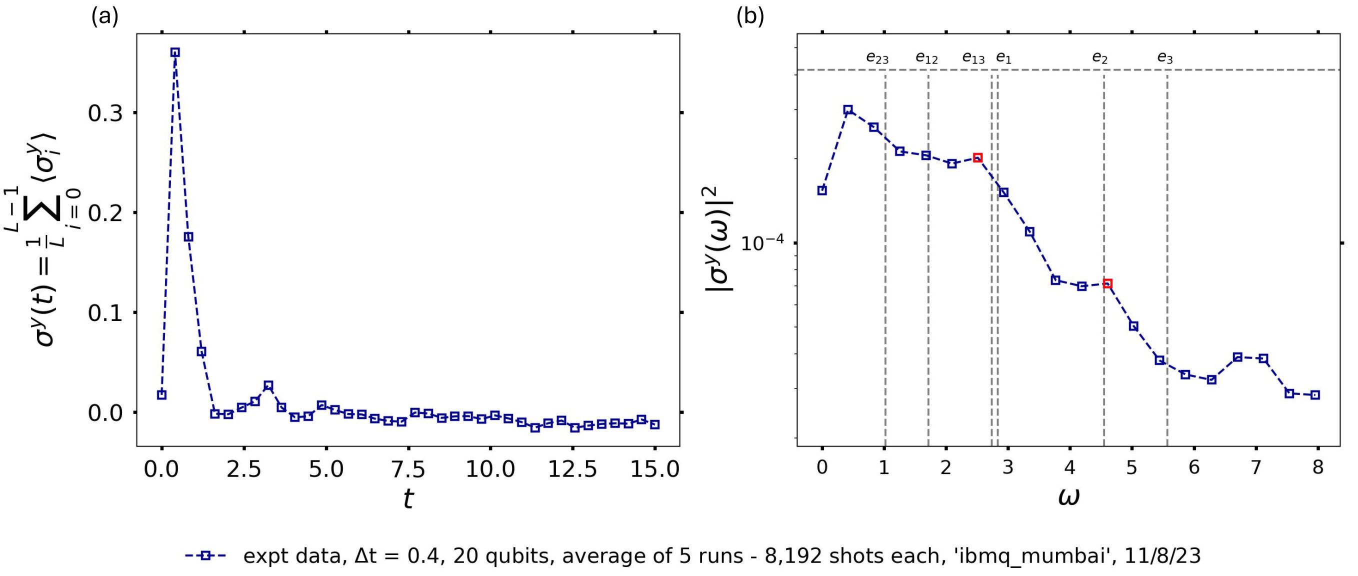

Supplementary Figure 4: Experimental results for the measurement of $\sigma^y(t)$, panel a), for the quench $(g,h):(0,0)\to(1.0,0.3)$ and the absolute value of the Fourier transform of the results, panel b). The results are an average of 5 experimental runs on IBM's Mumbai simulator, each with 8,192 shots. M3 and dynamical decoupling schemes were used to mitigate the effects of noise. The ratio between the marked maxima (red markers) is 1.833. Dashed blue lines are a guide for the eye.

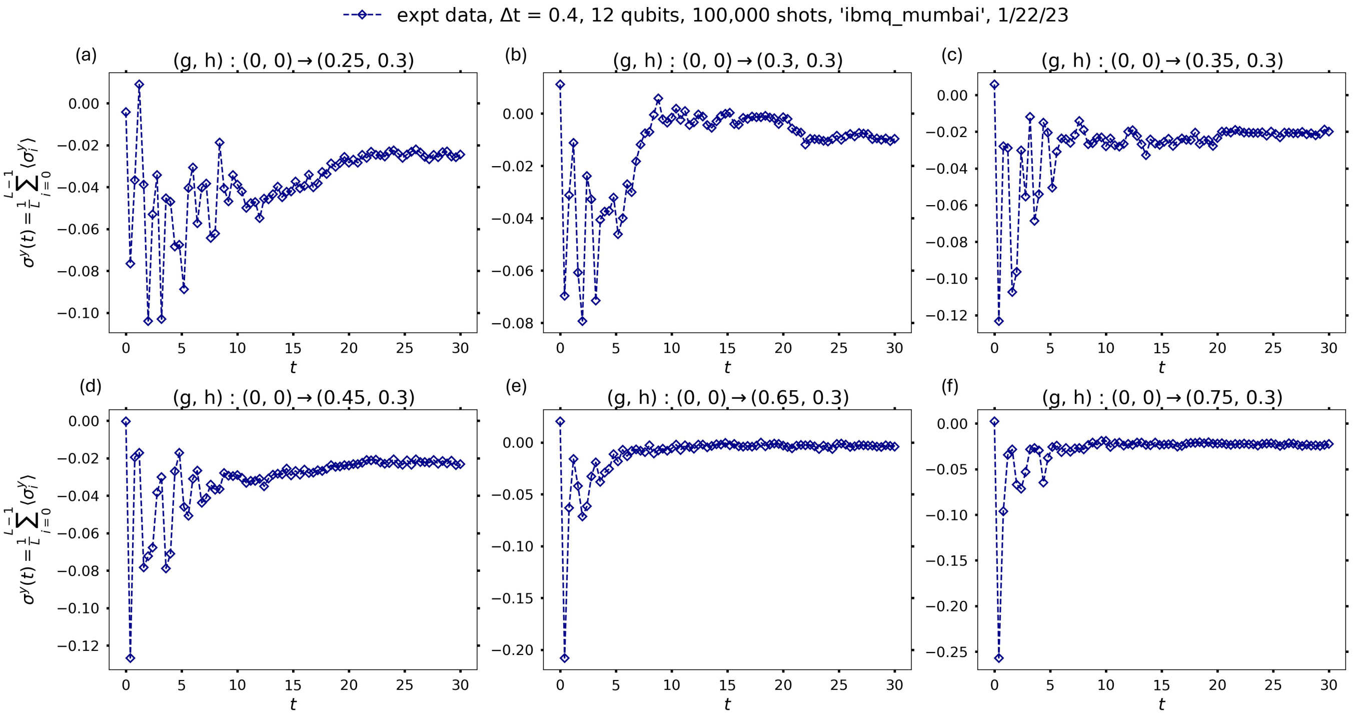

Supplementary Figure 5: Experimental results of the measurement of $\sigma^y(t)$ [panels a) - f)] for the quenches from $g=h=0$. The dynamical decoupling and M3 error mitigation techniques available Qiskit Runtime are used to mitigate the noise on the IBM Quantum Mumbai simulator. Dashed blue lines are a guide for the eye.

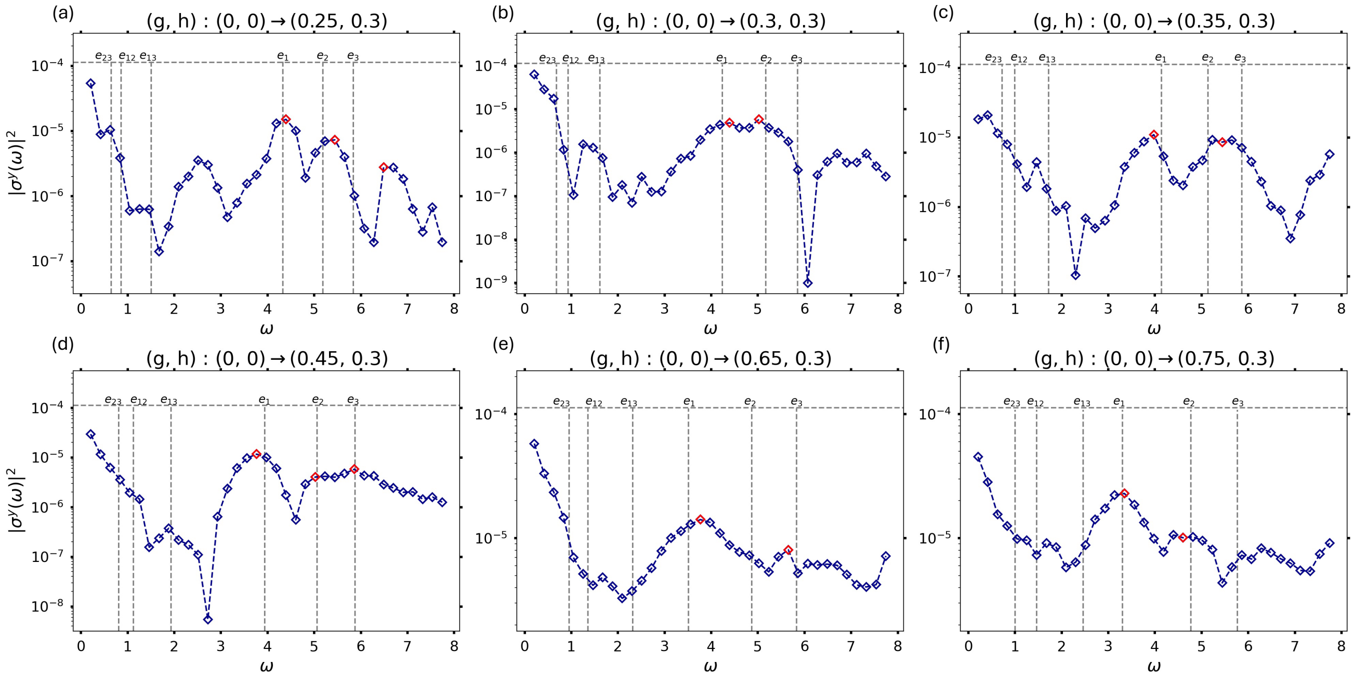

Supplementary Figure 6: Absolute value of the Fourier transform of the data shown in Figure 5 [panels a) - f)]. The different meson rest energies corresponding to the local maxima are marked in red. By locating the local maxima, error bars were added to Figure 3 of the main text with the value of $2\pi/t$ where $t = 30$. For the values $g = 0.35$ and $g = 0.75$, the error bars for e_2 are $4\pi/t$ since the location is not as clear. Dashed blue lines are a guide for the eye.

C. The XYZ Model with a transverse and longitudinal field

We start with the initial state $|\psi_0\rangle = |\uparrow\rangle^{\otimes L}$ when $J_x = J_y = h_x = h_z = 0$ and $L = 20$ with periodic boundary conditions. The system is time evolved with a unitary comprised of the second-order trotterization of the Hamiltonian:

$$H = -\sum_{i=0}^{N-1}\left(J_x\sigma_i^x\sigma_{i+1}^x + J_y\sigma_i^y\sigma_{i+1}^y + J_z\sigma_i^z\sigma_{i+1}^z\right) - \sum_{i=0}^{N-1} h_z\sigma_i^z - \sum_{i=0}^{N-1} h_x\sigma_i^x$$

We broke the unitary into the single-qubit operators, A, and two-qubit operators, B. The two-qubit operations are applied to the even and odd qubit indices to run the gates in parallel. The second order is applied to the single-qubit and even two-qubit operators to reduce errors caused by trotterization.

$$e^{-iHt} \approx \prod_{1}^{n} e^{-iA\frac{\delta t}{2}} e^{-iB_{even}\frac{\delta t}{2}} e^{-iB_{odd}\delta t} e^{-iB_{even}\frac{\delta t}{2}} e^{-A\frac{\delta t}{2}} \tag{2}$$

where, A and B are:

$$A = -\sum_{i=0}^{N-1}\left(h_z\sigma_i^z + h_x\sigma_i^x\right) \qquad B = -\sum_{i=0}^{N-1}\left(J_x\sigma_i^x\sigma_{i+1}^x + J_y\sigma_i^y\sigma_{i+1}^y + J_z\sigma_i^z\sigma_{i+1}^z\right)$$

The simulations were executed with two different optimization levels, 1 and 3, through the Qiskit transpiler. Optimization level 1 optimizes the circuit by collapsing adjacent gates, and level 3 optimizes with gate cancellation by commutativity rules and unitary synthesis. Level 1 is the closest to the exact results but has too many gates on the later time evolution circuits to execute on the available machines. Both optimization levels were run for the noiseless simulation and are included in Supplementary Figure 7 for comparison. During the transpilation, the dynamical decoupling sequence XYXY was added in the scheduling pass.

The experimental data was collected from `ibmq_mumbai` with the 20 qubit layout shown in Figure 1 of the main text. The experimental data shown in Supplementary Figure 7 is the average for 5 runs each with 8192 shots. The

results had the M3 readout error mitigation applied to the quasi-probabilities returned using the Qiskit IBM Runtime primitive, Sampler. The quasi-probabilities were converted to binary probabilities and used to calculate $\sigma^y(t)$. These results are then Fourier transformed, and the absolute value squared is taken to look for the rest energies (e_n) of the mesonic excitations.

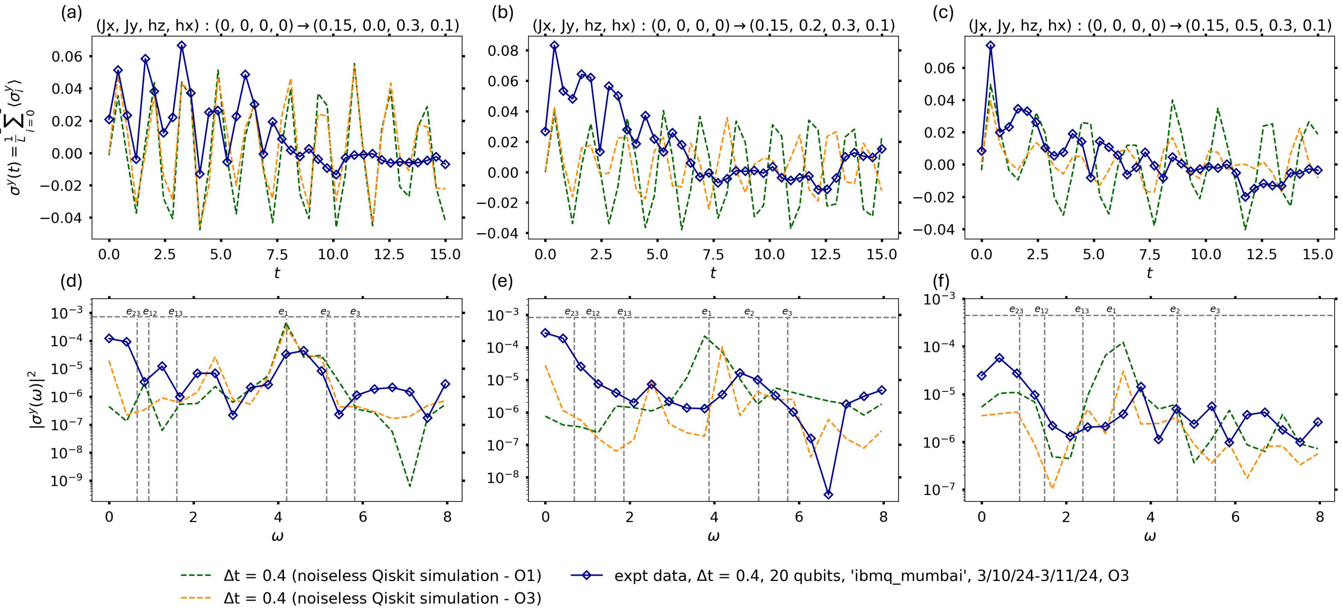

Supplementary Figure 7: XYZ meson spectroscopy simulation with experiment results for quench from $J_x = J_y = 0$, $h_x = h_z = 0$. The Qiskit M3 error mitigation and dynamical decoupling error suppression schemes were used to mitigate the effects of noise in the `ibmq_mumbai` simulator. (Top panels) $\sigma^y(t)$ results for different quench parameters with $L = 20$ qubits and periodic boundary conditions. The noiseless Qiskit simulations are performed with $\Delta t = 0.4$ with two different circuit optimization levels. Optimization level 1, O1, optimizes the circuit by simple adjacent gate collapsing. Optimization 3, O3, optimizes with gate cancellation by commutativity rules and unitary synthesis. The results are an average of 5 runs with 8192 shots each. The experimental data from `ibmq_mumbai` are shown as blue diamonds. (Bottom panels) The squared absolute values of the Fourier transform of $\sigma^y(t)$ are shown. The peaks correspond to the rest energies (e_n) of the mesonic excitations labeled by n and their differences (e_{mn}). The exact values are shown as gray dashed vertical lines.

D. The XY model in the presence of a longitudinal field

We start with the model that describes the quantum sine-Gordon model perturbed by a cosine potential with a twice the periodicity in the free-fermion limit, which is governed by the lattice Hamiltonian:

$$H = -\sum_{i=0}^{N-1} \left(J_x \sigma_i^x \sigma_{i+1}^x + J_y \sigma_i^y \sigma_{i+1}^y \right) - \sum_{i=0}^{N-1} h_x \sigma_i^x$$

with periodic boundary conditions. A mesonic state spectrum similar to the Ising case we studied can be observed after a global quench.

The quench protocol comprises the following steps:

1. *Initial state preparation:* The system is prepared in the ground state of the Hamiltonian with $h = 0, 0 \leq J_y$. In this work, we will restrict to initial product states: $|\psi_0\rangle = |\rightarrow\rangle^{\otimes L}$, *i.e.,* the case $J_y = 0, h = 0$, due to the simplicity of implementation on a quantum simulator. In this case, the initial state is prepared by simply applying a Hadamard gate on the canonical ground state of each spin.

2. *Trotterized time-evolution:* A global quench is performed to $h > 0$ with or without changing κ. The unitary evolution operator: $U = e^{-iHt}$ can be implemented on a classical computer either as a whole in exact simulations

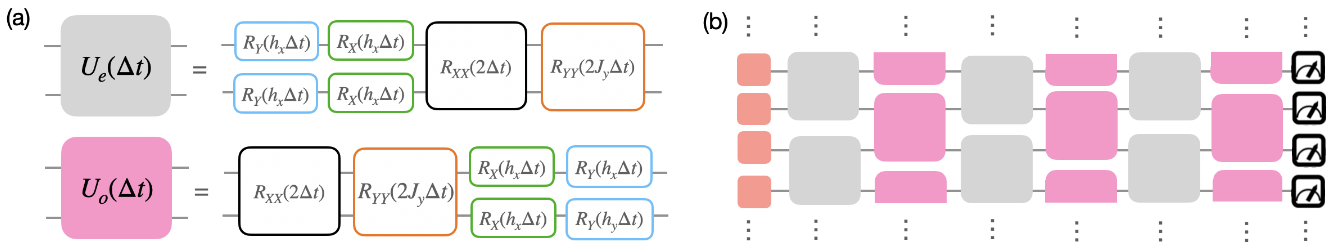

Supplementary Figure 8: (a) The decompositions of U_e and U_o in terms of single and two-qubit gates are shown. (b) Schematic of the trotterized unitary time-evolution generated by the Hamiltonian H. The action of the full unitary operator is decomposed into the unitary operators: U_e and U_o. At the end of the time-evolution, single qubit measurements are performed on the σ^x basis.

or as a sequence of two-site unitary operations in tensor-network computations, as shown in Fig. 8. For a quantum simulator, the unitary operator is decomposed into single and two-qubit gates; see below for more details.

E. Meson Energy in Zero Momentum Sector

To calculate the meson mass to get the static mesonic state, we want to diagonalize the Hamiltonian in the zero momentum sector (see for example, Ref. [1] for details). By writing the states in the σ^x basis (logical basis) as $|s_0, s_1, ..., s_{N-1}\rangle$, with $s_i = 0$ or 1. Thus a binary number from 0 to $2^N - 1$ can represent a basis without ambiguity. The transfer operator $T = e^{ipa}$ acts as $T|s_0, s_1, ..., s_{N-1}\rangle = |s_1, s_2, ..., s_{N-1}, s_0\rangle$. Thus a translational invariant state is defined as $T|\psi\rangle = |\psi\rangle$.

For an arbitrary basis vector $|a\rangle = |s_0, s_1, ..., s_{N-1}\rangle$, then $|\bar{a}\rangle = \sum_{n=0}^{N-1} T^n |a\rangle$ must be a translational invariant state. Defining an equivalent class such that if $|a\rangle = T^l |b\rangle$, then $|a\rangle$ and $|b\rangle$ are equivalent, $|\bar{a}\rangle$ from each class forms a complete basis for the zero momentum sector. The size of this equivalent class $p(a)$ is called the period since $T^p |a\rangle = |a\rangle$.

Using the translational symmetry, the matrix element of the Hamiltonian can be calculated as

$$H_{ab} = \langle \bar{b}| H |\bar{a}\rangle = \sqrt{\frac{p(a)}{p(b)}} \langle a| H |b\rangle \,. \tag{3}$$

Thus, one can diagonalize the Hamiltonian after finding all the matrix elements in the zero momentum sector. After diagonalizing the Hamiltonian in the zero-momentum sector, we calculated the energy $E_n = \langle n|H|n\rangle$ for all the eigenvectors $|n\rangle$.

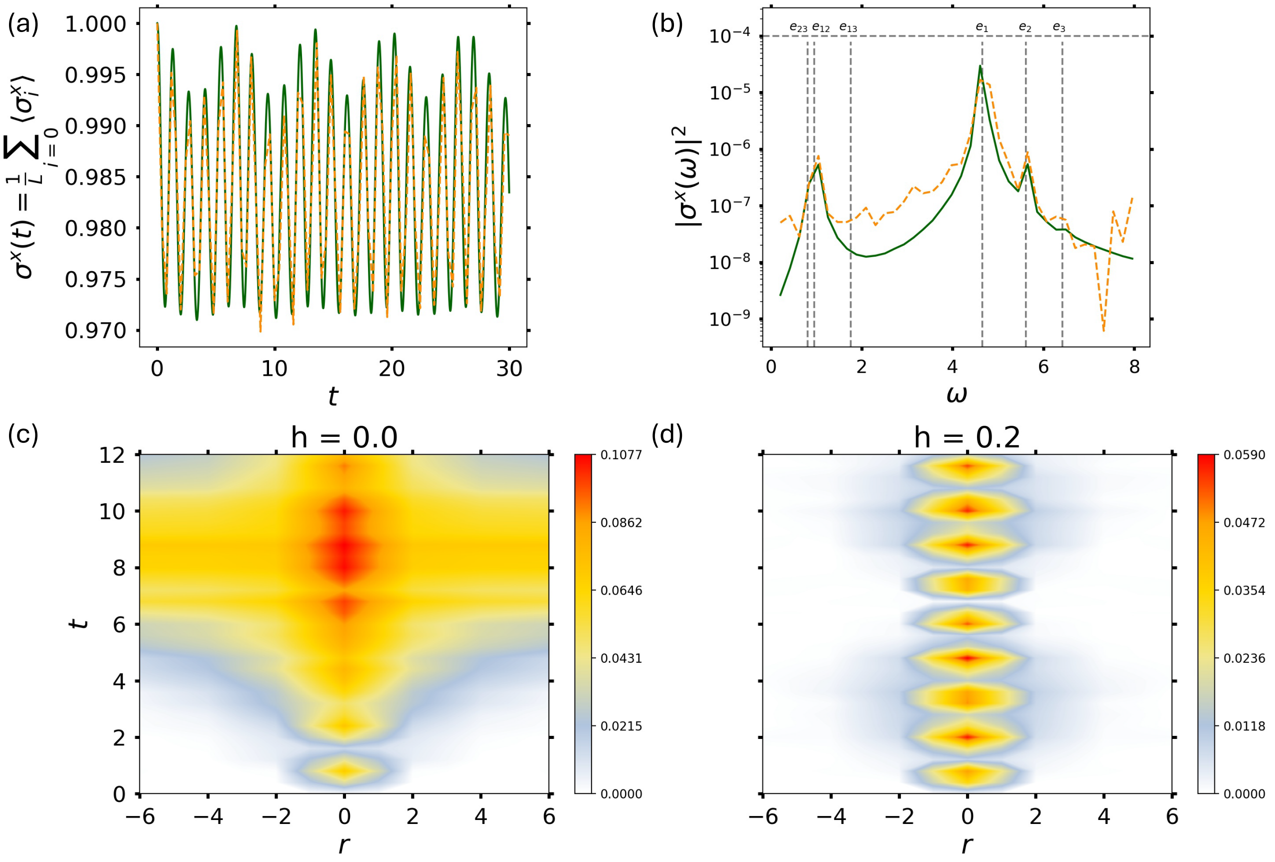

Supplementary Figure 9: (Top panels) Measurement results for $\sigma^x(t)$ and its Fourier transform $\sigma^x(\omega)$ after the quench with parameter $J_y = 0.2$ and $h_x = 0.2$. The dashed lines are meson mass calculated from exact diagonalization. The results obtained using an exact simulation of the time evolution of $L = 12$ qubits with $\Delta t = 0.01$ are shown with green solid lines.The noiseless numerical Qiskit simulation results are performed for $\Delta t = 0.4$ (orange dashed line). The peaks in the latter correspond to the rest energies (e_n), of the mesonic excitations labeled by n, and their differences (e_{mn}). The gray dashed lines correspond to the exact diagonalization results obtained in the zero momentum sector. (Bottom panels) Measurement results from noiseless Qiskit simulation for the correlation function $G_x(r,t) = \langle\sigma_i^x(t)\sigma_{i+r}^x(t)\rangle - \langle\sigma_i^x(t)\rangle\langle\sigma_{i+r}^x(t)\rangle$. The presence of the magnetic field clearly leads to the confinement of the domain wall excitations.

[1] A. W. Sandvik, Computational Studies of Quantum Spin Systems, AIP Conference Proceedings **1297**, 135 (2010), https://pubs.aip.org/aip/acp/article-pdf/1297/1/135/11407753/135_1_online.pdf.
